# Natural and Synthetic Variants of the Tricarboxylic Acid Cycle in Cyanobacteria: Introduction of the GABA Shunt into *Synechococcus* sp. PCC 7002

**DOI:** 10.3389/fmicb.2016.01972

**Published:** 2016-12-09

**Authors:** Shuyi Zhang, Xiao Qian, Shannon Chang, G. C. Dismukes, Donald A. Bryant

**Affiliations:** ^1^403C Althouse Laboratory, Department of Biochemistry and Molecular Biology, The Pennsylvania State University, University ParkPA, USA; ^2^Waksman Institute of Microbiology, Rutgers, The State University of New Jersey, PiscatawayNJ, USA; ^3^Department of Chemistry and Chemical Biology, Rutgers, The State University of New Jersey, PiscatawayNJ, USA; ^4^Department of Chemistry and Biochemistry, Montana State University, BozemanMT, USA

**Keywords:** photosynthesis, TCA cycle, GABA shunt, succinic acid semialdehyde, 2-oxoglutaric acid, *Synechococcus* sp. PCC 7002, *Synechocystis* sp. PCC 6803, cyanobacteria

## Abstract

For nearly half a century, it was believed that cyanobacteria had an incomplete tricarboxylic acid (TCA) cycle, because 2-oxoglutarate dehydrogenase (2-OGDH) was missing. Recently, a bypass route via succinic semialdehyde (SSA), which utilizes 2-oxoglutarate decarboxylase (OgdA) and succinic semialdehyde dehydrogenase (SsaD) to convert 2-oxoglutarate (2-OG) into succinate, was identified, thus completing the TCA cycle in most cyanobacteria. In addition to the recently characterized glyoxylate shunt that occurs in a few of cyanobacteria, the existence of a third variant of the TCA cycle connecting these metabolites, the γ-aminobutyric acid (GABA) shunt, was considered to be ambiguous because the GABA aminotransferase is missing in many cyanobacteria. In this study we isolated and biochemically characterized the enzymes of the GABA shunt. We show that *N*-acetylornithine aminotransferase (ArgD) can function as a GABA aminotransferase and that, together with glutamate decarboxylase (GadA), it can complete a functional GABA shunt. To prove the connectivity between the OgdA/SsaD bypass and the GABA shunt, the *gadA* gene from *Synechocystis* sp. PCC 6803 was heterologously expressed in *Synechococcus* sp. PCC 7002, which naturally lacks this enzyme. Metabolite profiling of seven *Synechococcus* sp. PCC 7002 mutant strains related to these two routes to succinate were investigated and proved the functional connectivity. Metabolite profiling also indicated that, compared to the OgdA/SsaD shunt, the GABA shunt was less efficient in converting 2-OG to SSA in *Synechococcus* sp. PCC 7002. The metabolic profiling study of these two TCA cycle variants provides new insights into carbon metabolism as well as evolution of the TCA cycle in cyanobacteria.

## Introduction

As mankind strives to lower anthropogenic carbon dioxide emissions, our ability to manipulate photosynthetic primary production is critical. Total worldwide primary production has been estimated to be 104.9 Gt C year^-1^, with roughly equal amounts arising from terrestrial and marine photosynthesis (Field et al., 1998). Cyanobacteria are an essential group of oxygenic photosynthetic organisms that are projected to play a pivotal role in CO_2_ sequestration and in supplying future renewable energy needs. Owing to their ease of genetic modification, many cyanobacteria have been engineered to improve CO_2_ capture and light utilization (Ducat et al., 2011; Lan and Liao, 2012; Yu et al., 2013). The capacity to improve these functions depends on how rapidly and completely the downstream steps can utilize the primary metabolic intermediates, thus fundamental studies are needed to understand key metabolic chokepoints and regulatory sites in carbon and energy metabolism (Krishnan et al., 2015b; Qian et al., 2015; Zhang et al., 2015).

**FIGURE 1:**
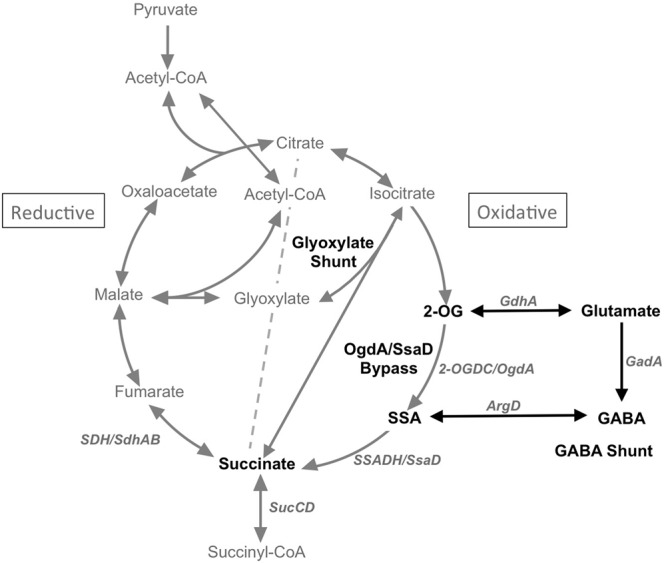
**Scheme showing the TCA cycle, the glyoxylate cycle and the GABA shunt.** 2-OG, 2-oxoglutarate; SSA, succinic semialdehyde; GABA, γ-aminobutyric acid; ArgD, GABA aminotransferase; GadA, glutamate decarboxylase; GdhA, glutamate dehydrogenase; 2-OGDC/OgdA, 2-oxoglutarate decarboxylase; SSADH/SsaD, succinic semialdehyde dehydrogenase.

The tricarboxylic acid (TCA) cycle is a central metabolic pathway that generates NADH reductant to power cellular growth, repair and homeostasis, as well as providing precursors for the biosynthesis of cellular components (Tang et al., 2011; Zhang and Bryant, 2014). Cyanobacteria were recently shown to have a complete but non-traditional TCA cycle (Zhang and Bryant, 2011). In this variation, 2-oxoglutarate (2-OG) is converted to succinate by 2-oxoglutarate decarboxylase (2-OGDC, OgdA) and succinate semialdehyde dehydrogenase (SSADH, SsaD) (**Figure 1**). The presence of this bypass in most cyanobacteria not only corrected a long-held misconception that these organisms have an incomplete TCA cycle due to the absence of 2-oxoglutarate dehydrogenase (2-OGDH), but also provided much useful knowledge illustrating the occurrence and physiological functions of TCA variants in these bacteria (Zhang and Bryant, 2011, 2014). Nonetheless, kinetic evidence for flux and regulation of this route remains poorly established. In particular, the involvement of this 2-OGDC/SSADH route under photoautotrophic and photomixotrophic growth conditions in *Synechococcus* sp. PCC 7002 (hereafter *Synechococcus* 7002) is so far unavailable. Flux-balance calculations have suggested that minimal flux occurs through the bypass reactions under photoautotrophic growth conditions (Hendry et al., 2016).

Other TCA cycle variants are known, including the glyoxylate shunt for acetate assimilation and the γ-amino-butyric acid (GABA) shunt that permits the sensing of nitrogen metabolite levels through glutamate. Based on comparative genomics and biochemical validation, Zhang and Bryant (2015) showed that the glyoxylate shunt only occurs in a few cyanobacteria. They characterized the two enzymes that complete this cycle, isocitrate lyase and malate synthase, in *Chlorogloeopsis fritschii* PCC 9212 and verified their respective activities. They also showed that this strain can grow in the dark on acetate as carbon and energy source. The introduction of the *aceAB* genes into *Synechococcus* 7002 conferred the ability to metabolize acetate in this strain and confirmed the role of the products of these genes in acetate metabolism via the glyoxylate shunt.

The existence of the GABA shunt in cyanobacteria is rather poorly understood. As shown in **Figure 1**, the GABA shunt includes four enzymes, glutamate dehydrogenase (GdhA), glutamate decarboxylase (GadA), GABA aminotransferase, and succinic semialdehyde dehydrogenase (SSADH/SsaD). The shunt results in the conversion of glutamate to succinate with GABA and succinic semialdehyde (SSA) as intermediates. However, according to comparative genomics, glutamate decarboxylase, which catalyzes the second step in the GABA shunt, is only present in a few cyanobacteria. Additionally, another key enzyme in the GABA shunt, the GABA aminotransferase is also missing in many cyanobacteria. Considering the metabolite links between the GABA shunt and the TCA cycle, both of which can convert 2-OG to succinate via SSA, it is likely that these two pathways are functionally connected. Indeed, it was reported in *Bradyrhizobium japonicum* that the GABA shunt is functional in a TCA cycle mutant lacking 2-OGDH (Green et al., 2000). A recent study has provided evidence that *N*-acetylornithine aminotransferase (ArgD) from *Synechocystis* sp. PCC 6803 (hereafter *Synechocystis* 6803) also functions as a GABA aminotransferase, which together with glutamate dehydrogenase (GdhA) and glutamate decarboxylase (GadA) form the GABA shunt in cyanobacteria (Xiong et al., 2014). However, biochemical validation of the putative bi-functional *N-*acetylornithine aminotransferase (ArgD) has not yet been demonstrated.

In this study, a protocol specific for cyanobacteria was developed to extract and analyze intracellular SSA from *Synechococcus* 7002. Our findings demonstrate the significant involvement of the OgdA/SsaD bypass under dark aerobic growth conditions in *Synechococcus* 7002, but suggest that there is much lower activity under photoautotrophic conditions. Additionally, we biochemically demonstrated that *N*-acetylornithine aminotransferase (ArgD) can also function as a GABA aminotransferase. Finally, the *gadA* gene, encoding glutamate decarboxylase from *Synechocystis* 6803, was expressed in *Synechococcus* 7002, which naturally lacks this enzyme. Intracellular metabolite concentrations were measured in this overexpression strain, and potential interactions between the resulting GABA shunt and the TCA cycle were investigated. The findings in this study validate the occurrence of three potential hybrid TCA cycle variants in some cyanobacteria and provide insights into pathway evolution as well as suggestions for future metabolic engineering targets.

## Materials and Methods

### Strains, Growth Conditions, and Culture Preparation

The strains used in this study, *Synechococcus* sp. PCC 7002 (WT7002) and *Synechocystis* sp. PCC 6803 (WT6803), were originally obtained from the Pasteur culture collection (PCC) (Rippka et al., 1979). Routine DNA manipulations were performed with *Escherichia coli* strain DH5α cells that were grown in Luria-Bertani (LB) medium. All *Synechococcus* 7002 strains were maintained in liquid medium A^+^ (Stevens et al., 1973), which contains 10 mM nitrate, and *Synechocystis* 6803 cells were grown in medium BG-11 (Rippka et al., 1979). The growth media were supplemented with the following antibiotics when appropriate: spectinomycin (50 μg ml^-1^), kanamycin (100 μg ml^-1^), gentamycin (20 μg ml^-1^), and/or erythromycin (20 μg ml^-1^). Unless noted, cultures were grown photoautotrophically under the following conditions: continuous irradiance of 120 μmol photons m^-2^ s^-1^ at 38°C with continuous sparging with 2% (v/v) CO_2_ in air. Two mutant strains lacking subunits of succinyl-CoA synthase (SucCD): SZ006 and SZ007 (**Table 1**) could not be grown under the specified high-CO_2_ sparging conditions. As a result, these two strains were grown by slowly bubbling cultures with 1% (v/v) CO_2_ in air. Growth of *Synechococcus* 7002 was measured by following changes in the optical density at 730 nm (OD_730 nm_) with a spectrophotometer.

**Table 1:** Plasmids and bacterial strains used in this study.

Plasmid or strain	Description	Reference
Plasmids		
pAQ1Ex	pGEM-7zf pMB1 vector backbone with *Synechococcus* sp. strain PCC 7002 pAQ1 flanking sites, Sp^R^	Xu et al., 2011
pAQ1Ex-*P_cpcBA_* [*gadA_6803_*]	pAQ1Ex *gadA* (from *Synechocystis* sp. strain PCC 6803) expression vector under control of *P_cpcBA_*, Sp^R^	This study
pAQ1Ex-*P_cpcBA_* [*argD_6803_*]	pAQ1Ex *argD* (from *Synechocystis* sp. strain PCC 6803) expression vector under control of *P_cpcBA_*, Sp^R^	This study
pAQ1Ex-*P_cpcBA_* [*argD_7002_*]	pAQ1Ex *argD* (from *Synechococcus* sp. strain PCC 7002) expression vector under control of *P_cpcBA_*, Sp^R^	This study
Strains
WT6803	Wild-type *Synechocystis* sp. strain PCC 6803	Rippka et al., 1979
WT7002	Wild-type *Synechococcus* sp. strain PCC 7002	Rippka et al., 1979
SZ001	WT7002 *ΔogdA*, Em^R^	This study
SZ002	WT7002 *ΔssaD*, Km^R^	This study
SZ003	WT7002 *ΔsdhB*, Km^R^	This study
SZ004	WT7002 *ΔsdhA*, Sp^R^	This study
SZ005	WT7002 *ΔsdhA ΔogdA ΔssaD*, Em^R^ Sp^R^	This study
SZ006	WT7002 *ΔsucCD*, Gm^R^	This study
SZ007	WT7002 *ΔsucCD ΔsdhB ΔogdA ΔssaD*, Em^R^ Gm^R^ Km^R^	This study
SZ008	WT7002 strain transformed with plasmid pAQ1Ex-*P_cpcBA_* [*gadA_6803_*], Sp^R^	This study
SZ009	SZ008 *ΔogdA*, Em^R^ Sp^R^	This study

To prepare cultures for metabolite analyses, all mutant strains were grown under the same photoautotrophic conditions as a WT7002 control sample until the cultures reached mid-exponential phase and had an OD_730 nm_ of ∼0.6. At this point, an aliquot of cell culture (2 mL) was collected, cells were rapidly harvested by vacuum filtration, and the cells were extracted for metabolomic analysis following the previously described protocol (Bennette et al., 2011). After being sampled for the photoautotrophic metabolomic analysis, cultures were quickly wrapped with aluminum foil and incubated without sparging in a dark growth chamber for 3 h at 38°C. The caps on the culture flasks were left loose so that the cultures inside would remain aerobic. After the 3-h dark aerobic incubation, cultures were sampled for dark aerobic metabolomic analysis following the same protocol (Bennette et al., 2011).

To prepare cultures for the ^13^C-labeling experiment under photomixotrophic conditions, the WT7002 strain was pre-acclimated to medium A^+^ supplemented with 5 mM glycerol. ^13^C_3_-labeled glycerol was used to conduct this experiment, because *Synechococcus* 7002 can metabolize exogenous glycerol but not glucose as a reduced carbon source (Shen and Bryant, 1995). Before the experiment, a WT7002 culture (100 mL) was grown photomixotrophically to an OD_730 nm_ of ∼1.0. An aliquot (500 μL) of the ^13^C_3_-labeled glycerol stock (1 M) was added immediately after the T_0_ sample (*t* = 0 s) was taken. Sampling continued for T_1_ (*t* = 0.50 min), T_2_ (*t* = 1 min), T_3_ (*t* = 10 min), T_4_ (*t* = 20 min), and T_5_ (*t* = 40 min). Immediately after sample T_5_ was taken, the culture vessel was wrapped in foil and placed in a dark growth chamber. The purpose of this switch was to identify the carbon source used by *Synechococcus* 7002 under dark aerobic conditions in the presence of an external reduced carbon source. Sampling was performed under dark aerobic conditions for T_6_ (*t* = 45 min), T_7_ (*t* = 60 min), and T_8_ (*t* = 90 min).

### Cloning, Protein Purification, and Identification

Open reading frames *sll1641* (*gadA*), encoding the glutamate decarboxylase, and *slr1022* (*argD*), encoding the *N-*acetylornithine aminotransferase of *Synechocystis* 6803, as well as open reading frame SYNPCC7002_A0326 (*argD*), encoding the *N-*acetylornithine aminotransferase of *Synechococcus* 7002, were amplified by polymerase chain reaction (PCR) with Phusion DNA polymerase (New England Biolabs, Ipswich, MA, USA) and cloned into plasmid pAQ1Ex-*P_cpcBA_* (Xu et al., 2011). Primer set GADF-GADR was used to amplify *gadA*; primer set 6803ArgDF-6803ArgDR was used to amplify *Synechocystis* 6803 *argD* (*argD*_6803_); and primer set 7002ArgDF-7002ArgDR was used to amplify *Synechococcus* 7002 *argD* (*argD*_7002_) (Supplementary Table S1). An *N-*terminal His_10_-tag was introduced into each of the enzymes to facilitate subsequent protein detection and purification.

The resulting plasmids pAQ1Ex-*P_cpcBA_*[*gadA*_6803_], pAQ1Ex-*P_cpcBA_*[*argD_(6803)_*], and pAQ1Ex-*P_cpcBA_*[*argD*_7002_] (**Table 1**) were transformed into *E. coli* strain DH5-α and verified by DNA sequencing. Cells were grown overnight in 1 L of Luria-Bertani medium containing 50 μg gentamycin ml^-1^, harvested by centrifugation at 4°C at 5,000 × *g*, and washed once with 50 mM Tris-HCl buffer, pH = 8.0. Cells were disrupted by three passages through a chilled French pressure cell operated at 138 MPa. Soluble lysates were obtained by centrifugation at 20,000 × *g* for 30 min, and were loaded onto a Ni^2+^-NTA affinity resin (Goldbio, St. Louis, MO, USA). The column was pre-equilibrated with 10 mM imidazole in 50 mM Tris-HCl, pH 8.0 and was washed with 30 mM imidazole in 50 mM Tris-HCl, pH 8.0, 300 mM NaCl. Proteins were eluted stepwise with 50, 100, 150, 200, and 250 mM imidazole in 50 mM Tris-HCl, pH 8.0, 300 mM NaCl. Fractions containing the recombinant proteins were analyzed by polyacrylamide gel electrophoresis in the presence of sodium dodecylsulfate (SDS-PAGE) (Shen and Bryant, 1995), and purified proteins were concentrated by ultrafiltration using Centriprep columns (Millipore, Billerica, MA, USA). Proteins were further analyzed by SDS-PAGE and immunoblotting with commercial polyclonal rabbit antibodies (Rockland, Limerick, PA, USA) to the poly-His_10_ tag. Each protein was judged to be >90% pure and was also positively identified by tryptic peptide mass fingerprinting as previously described (Zhang and Bryant, 2011). Consistent with the results from mass spectrometry, no contaminating proteins were identified.

### Enzymatic Assays

For enzyme assays with *Synechocystis* 6803 glutamate decarboxylase (GadA_6803_ = the product of open reading frame sll1641), the reaction mixture (0.2 ml) contained 1 mM glutamate, 50 mM K-phosphate, pH 4.5 and purified enzyme (50 μg); control reaction mixtures were treated identically but without the addition of the purified enzyme. The reaction mixtures were incubated at room temperature (RT) for 1 h, and the GABA produced was detected by using Edman’s reagent as previously described with slight modifications (Tank and Bryant, 2015). An aliquot (30 μl) of the reaction mixture was transferred to a glass test tube and dried by flushing with nitrogen. The dried sample was dissolved in coupling solution (100 μL; mixture containing 50% acetonitrile, 25% pyridine, 10% triethylamine and 15% water, v/v/v/v), and the resulting solution was dried again. The dried sample was redissolved in coupling solution (100 μL), after which 5 μL of phenylisothiocyanate (PITC; Edman’s reagent) was added to the solution, and the coupling reaction was allowed to proceed for 5 min at RT. After the reaction was complete, the liquid sample was dried and the resulting pellet was dissolved in analysis solvent [250 μl, 7:2 (v/v) mixture of HPLC-grade water and acetonitrile]. An aliquot (20 μL) of the solution was injected onto a Shimadzu LC-20AB HPLC system equipped with 254-nm UV detector SPD-20A. Components in the solution were separated on a Kinetex 5-μm C18 100 Å column (15 cm × 4.6 174 mm ID) protected by a SecurityGuard ULTRA cartridge UHPLC C18 for 4.6-mm ID columns 175 (Phenomenex, Torrance, CA, USA). The HPLC analysis method consisted of a 2-solvent gradient (solvents A and B) developed over a 20-min period with a flow rate of 0.5 ml min^-1^ at 30°C. The initial condition was 100% solvent A (0.14 M sodium acetate, pH 6.2 containing 0.5 mM triethanolamine), which decreased over 10 min to 82.5% and from 10 to 15 min to 0%. Solvent B was a 40:60 (v/v) mixture of HPLC-grade water and acetonitrile. The elution times and concentrations of substrates and products were determined by comparison of results obtained from analyses of individual standard compounds with the same procedure.

For enzyme assays with *N-*acetylornithine aminotransferase (ArgD), the reaction mixture (0.2 ml) contained 1 mM L-ornithine, 1 mM 2-oxoglutarate, 50 mM K-phosphate, pH 9 and the purified enzyme (50 μg) from *Synechocystis* 6803 (ArgD_6803_) or *Synechococcus* 7002 (ArgD_7002_). Reaction mixtures were incubated at RT for 1 h, and an aliquot (30 μl) of the reaction mixture was analyzed using the same procedure as described for the characterization of glutamate decarboxylase to detect product formation. Control experiments were performed similarly but without the addition of the purified enzyme. The elution times and concentrations of substrates and products were determined by comparison of results obtained from analyses with purified individual standard compounds.

For enzyme assays with GABA aminotransferase (ArgD), the reaction mixture (0.2 ml) contained 1 mM GABA, 1 mM 2-OG, 50 mM K-phosphate, pH 9 and the purified enzyme (50 μg) from *Synechocystis* 6803 (ArgD_6803_) or *Synechococcus* 7002 (ArgD_7002_). The mixture was incubated at RT for 1 h, and an aliquot (30 μl) of the reaction mixture was analyzed using the same procedure as described in the enzyme characterization of glutamate decarboxylase to detect product formation. Control experiments were performed similarly but without the addition of the purified enzyme. The elution times and concentrations of substrates and products were determined by comparison of results obtained from analyses of individual standard compounds.

### Mutant Construction and Segregation

The coding sequences of *ogdA* (SynPCC7002_A2770), *ssaD*(SynPCC7002_A2771), *sdhA* (SynPCC7002_A2569), *sdhB* (SynPCC7002_A1094), or the *sucCD* operon (SynPCC7002_A0890 and SynPCC7002_A0891) were deleted and replaced by a DNA cassette that confers specific antibiotic resistance to produce the corresponding mutant strains of *Synechococcus* 7002 using the homologous recombination method (Frigaard et al., 2004) (see **Table 1**; Supplementary Figure S1A). In addition, the *ogdA ssaD* operon (SynPCC7002_A2770 SynPCC7002_A2771) was deleted in mutant strain SZ004 to generate the triple mutant strain SZ005; and *sdhB* (SynPCC7002_A1094) as well as the *ogdA ssaD* operon (SynPCC7002_A2770 SynPCC7002_A2771) were deleted in mutant strain, SZ006, to generate the quadruple mutant, SZ007. Complete segregation of alleles was verified in all cases by PCR with template DNAs derived from the wild-type and mutant strains (see Supplementary Figures S1B–I). The primers used for PCR analyses are listed in Supplementary Table S1. The mutant strains were also verified by DNA sequencing of the resulting PCR amplicons.

In order to construct a *Synechococcus* 7002 strain expressing the glutamate decarboxylase (GadA_6803_), the pAQ1Ex-*P_cpcBA_* [*gadA*_6803_] plasmid was transformed into wild-type *Synechococcus* 7002 as previously described (Frigaard et al., 2004). The presence of the *gadA*_6803_ gene in the resulting strain, denoted SZ008, was confirmed by PCR using primer set GADF-GADR. Immunoblotting was also performed with commercial antibodies (Rockland) to the His_10_-tag as previously described (Shen and Bryant, 1995) to confirm expression of the recombinant glutamate decarboxylase (Supplementary Figure S2).

To investigate further the metabolic correlation between the GABA shunt and the TCA cycle in cyanobacteria, the gene encoding 2-OGDC (*ogdA*; SYNPCC7002_A2770) was deleted and replaced by a DNA fragment encoding an antibiotic resistance gene (*aphAII*, kanamycin resistance), and the resulting strain was denoted as SZ009. Primer sets A2770D1–A2770D2 and A2770D3–A2770D4 (Supplementary Table S1) were used to amplify the upstream and downstream regions of *ogdA*. Transformation and selection were performed as previously described (Frigaard et al., 2004). Full segregation of the deletion of SYNPCC7002_A2770 was verified by PCR by comparing the products of template DNAs from both the wild-type and the mutant strains using primer set A2770D1–A2770D4 (Supplementary Figure S2). The resulting PCR amplicons were also verified by DNA sequencing.

### Intracellular Metabolites Analyses

Metabolite extraction and quantification were performed as previously described (Bennette et al., 2011). Briefly, an aliquot of cell culture (2 mL) was sampled and rapidly vacuum-filtered on a 0.45-μm Nylon membrane filter under dark conditions. The cells adhering to the membrane were immediately quenched in 1.8 mL of ice-cold buffer [80:20 (v/v) MeOH/H_2_O] in Petri dishes and incubated for 20 min at -20°C. Cell material was then scraped off from the filters, and the solvent and cell material suspension was centrifuged at 14,000 × *g* at 4°C for 5 min. The supernatant was removed and stored at -80°C. An aliquot of the solvent extract (120 μL) was vacuum-dried (Labconco Centri-Vap Concentrator), and the pellet was resuspended in LC–MS grade water (40 μL) for analysis. The LC–MS analysis system and methods were described previously (Bennette et al., 2011). Extracellular metabolite profiles were analyzed by HPLC according to a previously described protocol (Kumaraswamy et al., 2013).

### Determination of Intracellular SSA in *Synechococcus* 7002

Wild-type *Synechococcus* 7002 cultures were grown to OD_730nm_ = ∼0.6, and aliquots of the culture (15 mL) were collected before and after incubation under dark aerobic conditions for 3 h. The cells in the culture aliquots were pelleted by centrifugation at 9600 × *g* at 4°C for 15 min, and the supernatant was discarded. Cell pellets were resuspended in ice-cold, autoclaved water (500 μL), and the suspensions were sonicated 15 times each over ice (with each pulse lasting 10 s) using a Fisher Scientific Model FB-505 sonicator (amplitude, 40%). The sonicated samples were centrifuged at 14,000 × *g* at 4°C for 5 min. Supernatants (500 μL per sample) were collected and transferred into clean microcentrifuge tubes.

Because SSA is unstable in air (Wermuth, 1979), SSA extracts from cyanobacteria cannot be analyzed by the previously described metabolite isolation method (Bennette et al., 2011), which has an evaporation and concentration step in air. By adapting an SSA derivatization method that was originally used to quantify SSA in liquid samples of human urine (Struys et al., 2005), it is possible to detect and quantify intracellular SSA in the cyanobacterium *Synechococcus* 7002. SSA was derivatized with 2,4-dinitrophenylhydrazine (DNPH) and detected by LC–MS (Struys et al., 2005). Briefly, DNPH (50 μL of a 3.1 mg mL^-1^ stock solution prepared in 2.0 M HCl) was added to an aliquot (500 μL) of sample supernatant or SSA calibration standard (concentrations 0, 0.01, 0.05, 0.1, 0.5, and 1 mM), and the reaction mixtures were incubated for 15 min at RT. The SSA derivatives were extracted with ethyl acetate (3.0 mL), and the organic fractions were transferred to clean glass tubes and dried with a stream of nitrogen gas. The dry residue was dissolved in 100% methanol (125 μL), and an aliquot (10 μL) of the liquid sample was injected into an LC–MS/MS system for analysis. Mass transitions were set to be *m/z* –281.1 →*m/z*-182.1 and *m/z* -285.1 →*m/z*-182.1 for ^13^C_0_-SSA (M_0_) and ^13^C_4_-SSA (M_4_), respectively.

### O_2_ Evolution Rate, Dark Respiration Rate, and Measurement of Chlorophyll *a* Concentrations

Liquid cultures of WT7002 and mutant strains were grown to mid-exponential phase (OD_730 nm_ = ∼0.6), and cells were harvested to measure their maximal O_2_ evolution rates under saturating red light (∼750 μmol photons m^-2^ s^-1^) and their dark respiration rates. These rates were measured using Clark-type electrodes, employing both commercial (Hansatech) and home-built electrodes (Krishnan et al., 2015a). Sodium bicarbonate (25 mM) was added to the cell suspensions prior to analysis in order to provide excess C_i_ source to act as the electron acceptor for the photoreactions. Chlorophyll (Chl) *a* concentrations were measured according to the colorimetric method previously described (Sakamoto and Bryant, 1997).

## Results

### Identification of SSA-DNPH Derivatives

**FIGURE 2:**
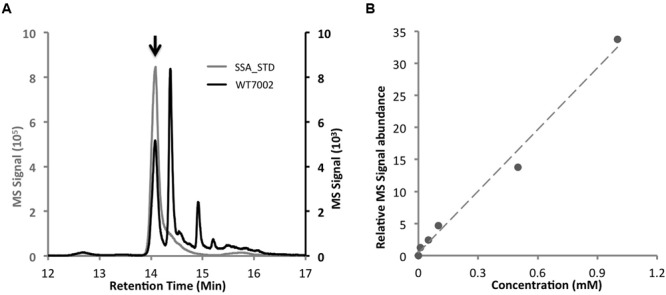
**LC–MS method to determine intracellular SSA contents. (A)** Comparison of chromatograms of WT7002 (black line) and 2.5 mM SSA standard (gray line). Black arrow indicates the peak of SSA. **(B)** Standard curve of SSA within the concentration range of 0 to 1.0 mM, *R*^2^ = 0.99.

Based on a comparison of the LC chromatograms of extracts of the WT7002 strain with a 2.5 mM SSA standard (**Figures 2A,B**), the retention time of SSA-DNPH derivatives was determined to be 14.1 min. The calibration curve was linear over the range 0–1.0 mM for SSA-DNPH derivatives, with a linear regression (R^2^) of 0.99 (**Figure 2B**). Deconvolution of the MS/MS spectra of SSA-DNPH showed an intense fragment at *m/z* -182.1, which corresponds to the loss of the DNPH group from SSA-DNPH.

### ^13^C-Labeling of SSA under Light and Dark Conditions

**FIGURE 3:**
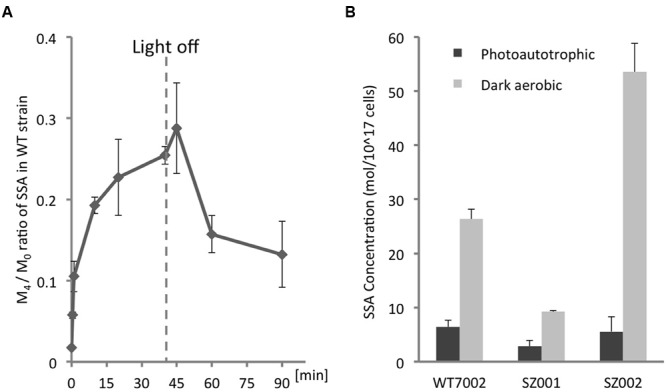
**Quantification of intracellular SSA. (A)**^13^C-labeling kinetics of SSA under photomixotrophic conditions. The dashed line indicates the time point when the culture was switched from light to dark. **(B)** Intracellular SSA contents of WT7002 and strains SZ001, and SZ002 under photoautotrophic and dark aerobic conditions. The results are mean values of three biological replicates and error bars represent the standard deviations.

The level of ^13^C-labeling of intracellular SSA was determined by measuring the [^13^C_4_-SSA]/[^13^C_0_-SSA] ratios (M_4_ /M_0_) over time (**Figure 3A**). Other SSA forms with 1, 2, or 3 ^13^C-labeled carbons (M_1_, M_2_, and M_3_) were assayed as well, and it was determined that ^13^C_4_-SSA was the clearest and free from interference. Starting with the WT7002 strain under photoautotrophic conditions, the M_4_/M_0_ value increased immediately after addition of ^13^C-labeled glycerol (**Figure 3A**) and attained a steady-state value of 0.25 at 40 min. The culture was switched to dark aerobic conditions at 40 min and the M_4_/M_0_ value slowly decreased over the next 50 min to a value of 0.15 after the light-to-dark transition.

### Intracellular Metabolite Analysis of TCA Cycle Mutants

The differences in SSA content among WT7002 and two single mutant strains, SZ001 and SZ002 (see **Table 1**), were monitored under both photoautotrophic and dark aerobic conditions (**Figure 3B**). Under photoautotrophic conditions, WT7002 had ∼2-fold more SSA than the SZ001 strain, but had a comparable SSA content to the SZ002 strain. However, after 3 h of dark aerobic incubation and compared to WT7002, the SZ002 strain accumulated ∼2-fold more SSA while the SZ001 mutant had 2.5-fold lower SSA. These results confirm that 2-OGDC (OgdA; product of SynPCC7002_A2770) is functional under both photoautotrophic and respiratory conditions.

**FIGURE 4:**
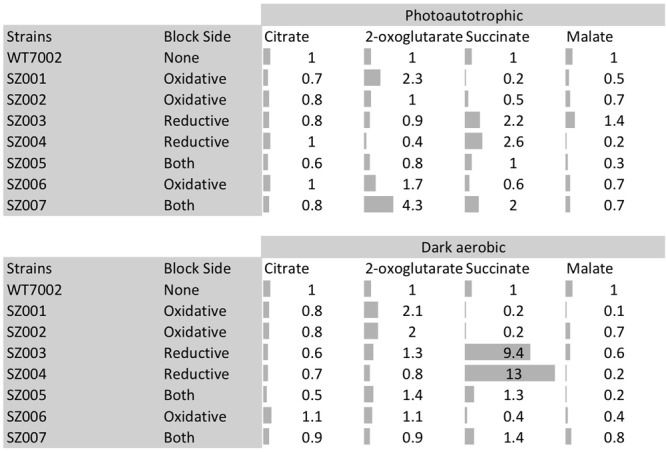
**Metabolites profiling of TCA cycle metabolites in mutant strains.** Category “block side” refers to the side of the TCA cycle (reductive side or oxidative side) that hypothetically prevent the synthesis of succinate due to the corresponding mutation(s). Gray bars indicate the fold changes of these metabolites compared to WT7002 levels.

Intracellular contents of four TCA cycle metabolites (citrate, 2-OG, succinate, and malate) in the mutant strains were compared to those in the WT7002 strain (**Figure 4**). Mutants were categorized into three groups according to a terminology as follows: oxidative, reductive and “both branches.” These terms indicate the location of the missing enzyme(s) in the TCA cycle, using citrate-succinate as the dividing line (dashed line in **Figure 1**, right side: oxidative; left side: reductive). In the two mutant strains in the “oxidative” group, i.e., SZ001, and SZ002 and compared to the levels in the WT7002 strain, the succinate content downstream of these enzymes was significantly reduced under both light and dark conditions. Under photoautotrophic conditions, the 2-OG content in the strain lacking 2-OGDC (OgdA) increased 2.3-fold, as expected, while it remained at a similar level as in the WT7002 strain in the SZ002 mutant strain lacking SSADH. After a 3-h dark aerobic incubation, the 2-OG content in both mutant strains was twofold higher than in strain WT7002. In the succinyl-CoA synthase (*sucCD*) mutant SZ006, the succinate and malate contents were both reduced to ∼60% of the levels in strain WT7002 under photoautotrophic conditions, and the levels of these compounds decreased further to ∼40% of the levels in WT7002 under dark aerobic conditions.

In the two mutant strains designated as “reductive branch,” SZ003 and SZ004, succinate contents were 2.2- and 2.6-fold higher than in strain WT7002, respectively, under photoautotrophic conditions. Succinate levels in these two strains were substantially higher, 9.4- and 13.0-fold higher, respectively than the in strain WT7002, under dark aerobic conditions. The content of the downstream metabolite, malate, was significantly reduced in the SZ004 strain under both conditions, but it remained at similar levels to WT7002 in the SZ003 strain. In the triple mutant strain, SZ005, designated as “both branches,” 2-OG and succinate remained at similar levels as for WT7002, while the malate content decreased under both conditions. In the quadruple mutant strain, SZ007, designated as “both branches,” 2-OG and succinate were 4.3- and 2.0-fold higher than in WT7002, respectively, under photoautotrophic conditions. The increased pool sizes diminished when the mutant strains were incubated under dark aerobic conditions. In summary, the pattern of the pool size changes is consistent with the operation of a cycle catalyzed by enzymes.

### Growth Phenotypes and Succinate Levels in Mutant Strains

Four single mutant strains (SZ001, SZ002, SZ003, SZ004) showed reduced photoautotrophic growth rates compared to WT7002 (**Table 2**). The Chl *a* contents and O_2_ evolution rates of these mutant strains were all lower than those of WT7002 (**Table 2**). Dark respiration rates of the SZ003 and SZ004 mutants, which lack a functional succinate dehydrogenase (SDH) enzyme complex, were reduced more than 44% compared to WT7002. Disruptions in the SSA bypass of the oxidative branch caused little or no respiratory deficiency in the corresponding mutant strains (SZ001 and SZ002).

**Table 2:** Growth rates, Chl *a* contents, oxygen evolution and respiration rates of mutant strains.^1^

Strain	Doubling time (h)	Chl *a* content (μg/10^8^ cells)	O_2_ evolution rate (nmol/μg Chl *a*/h)	O_2_ respiration rate (nmol/10^8^ cells/h)
WT7002	3.2 ± 0.3	12.1 ± 0.5	871 ± 35	892 ± 68
SZ001	3.8 ± 0.2	10.2 ± 0.3	654 ± 15	685 ± 2
SZ002	3.6 ± 0.2	9.6 ± 0.4	703 ± 31	798 ± 34
SZ003	3.9 ± 0.1	9.0 ± 0.3	689 ± 29	537 ± 3
SZ004	3.7 ± 0.1	8.8 ± 0.5	586 ± 23	482 ± 25
SZ008	3.7 ± 0.3	8.3 ± 0.5	713 ± 36	582 ± 82
SZ009	6.9 ± 0.4	9.9 ± 0.5	369 ± 46	592 ± 92

*^1^The data shown are averages and standard deviations from three biological replicates.*

Under both photoautotrophic and dark aerobic respiratory conditions, the succinate pool size increased when either of the succinate dehydrogenase subunits was deleted and decreased when the SSA bypass route was disrupted (**Figure 4**). These results indicate that, under photoautotrophic and dark aerobic conditions, (1) removal of either subunit of the succinate dehydrogenase complex abolishes the enzyme activity of succinate dehydrogenase; (2) the SSA route is functional *in vivo*; and (3) the oxidative TCA cycle (operation in the clock-wise direction of **Figure 1**) together with other potential alternative succinate synthesis pathways are dominant over the reductive TCA branch (counter clock-wise direction of **Figure 1**) in the synthesis of succinate. In agreement with the present findings, succinate accumulation increased ∼2- to 3-fold in *Synechocystis* 6803 when 2-OG was added to the growth medium of an *sdhB* mutant (Cooley et al., 2000; Cooley and Vermaas, 2001).

### GABA Shunt Enzymatic Assays

**FIGURE 5:**
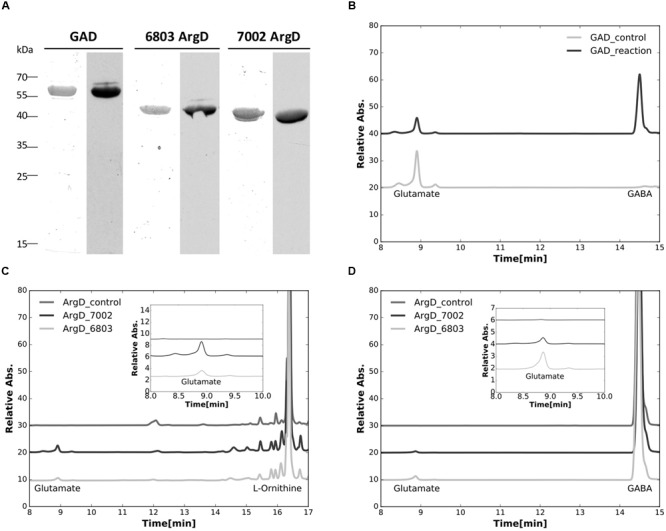
**Characterizations of purified recombinant proteins. (A)** Purified recombinant proteins of the glutamate decarboxylase (GadA), and the *N*-acetylornithine aminotransferase from both *Synechocystis* sp. PCC 6803 (ArgD_6803_) and *Synechococcus* sp. PCC 7002 (ArgD_7002_) were analyzed. Left lanes for each enzyme were stained with Coomassie blue and right lanes were detected by immunoblotting with antibodies to the poly-[His]_10_-tag. **(B)** HPLC analysis showing the conversion of glutamate to GABA, catalyzed by the purified glutamate decarboxylase (GAD_reaction). **(C)** HPLC analysis showing the formation of glutamate from L-ornithine catalyzed by the purified *N*-acetylornithine aminotransferase from both *Synechocystis* sp. PCC 6803 (ArgD_6803) and *Synechococcus* sp. PCC 7002 (ArgD_7002). **(D)** HPLC analysis showing the formation of glutamate from GABA catalyzed by the purified *N*-acetylornithine aminotransferase from both *Synechocystis* sp. PCC 6803 (ArgD_6803) and *Synechococcus* sp. PCC 7002 (ArgD_7002), demonstrating that *N*-acetylornithine aminotransferase can also function as GABA aminotransferase. Insets in **(C,D)** represent the enlarged parts of the elution curves from 8 to 10 min to illustrate the changes of the glutamate peaks observed more clearly. It should be noted that the large differences in peak heights occur because the different compounds have very different molar extinction coefficients at the detection wavelength. Control experiments in all of these assays were performed the same way without recombinant proteins added.

The genes encoding glutamate decarboxylase (*gadA*_6803_) and *N-*acetylornithine aminotransferase (*argD*_6803_) of *Synechocystis* 6803, as well as the *N-*acetylornithine aminotransferase (*argD*_7002_) of *Synechococcus* 7002 were successfully expressed and the products purified from *E. coli* as *N-*terminally poly-[His]_10_-tagged proteins. The purified proteins were positively immunoreactive with commercial antibodies to the poly-[His]_10_ tag (**Figure 5A**), and were also identified by tryptic peptide mass fingerprinting (data not shown).

The enzymatic activity of glutamate decarboxylase, GadA_6803_, protein was characterized by confirming the consumption of substrate, glutamate, and parallel formation of GABA (**Figure 5B**). This result establishes that the *gadA* gene encodes glutamate decarboxylase and this protein catalyzes the conversion of glutamate to GABA. The enzymatic activity of *N-*acetylornithine aminotransferase (ArgD_6803_) was also assayed. When the protein product from *argD*_6803_ or *argD*_7002_ was incubated with L-ornithine and 2-OG, glutamate was produced (**Figure 5C**). This shows that these two ArgD proteins have *N-*acetylornithine aminotransferase activity. Because it has been suggested that *N-*acetylornithine aminotransferase could also function as GABA aminotransferase (Xiong et al., 2014), we investigated this postulated activity. When the protein product from *argD*_6803_ or *argD*_7002_ was incubated with GABA and 2-oxoglutarate, glutamate was produced in the reaction mixture (**Figure 5D**). These biochemical results established that *N-*acetylornithine aminotransferase is a bifunctional enzyme that has both *N-*acetylornithine aminotransferase and GABA aminotransferase activities.

### Construction of Glutamate Decarboxylase Expression Strains

The foregoing biochemical results validated the *in vitro* enzymatic activities of the postulated GABA shunt, which is clearly functional in some cyanobacteria, in particular *Synechocystis* 6803. However, the apparent absence of glutamate decarboxylase in most cyanobacteria (e.g., *Synechococcus* 7002) suggests that the GABA shunt is not universally present in cyanobacteria (Xiong et al., 2014). To investigate the biochemical role of the GABA shunt in cyanobacterial metabolism, a strain of *Synechococcus* 7002 expressing glutamate decarboxylase was constructed (SZ008), in which the glutamate decarboxylase (*gadA*_6803_) from *Synechocystis* 6803 was expressed under control of the strong *P_cpcBA6803_* promoter (Xu et al., 2011; Zhou et al., 2014). The presence of the plasmid and the incorporation of the *gadA*_6803_ gene into *Synechococcus* 7002 were verified by PCR amplification of the *gadA*_6803_ gene (Supplementary Figure S2A) and DNA sequencing of the amplicon further confirmed the construction of strain SZ2008. Successful expression of glutamate decarboxylase was confirmed by immunoblotting using antibodies against His_10_-tag, as shown in Supplementary Figure S2B. The gene encoding 2-oxoglutarate decarboxylase (*ogdA*; SYNPCC7002_A2770) was deleted in strain SZ008 to generate strain SZ009. Complete segregation of the mutant alleles was confirmed by PCR using primer set A2770D1-A2770D4 as shown in Supplementary Figure S2C. Compared to the WT7002 strain, strain SZ008 had a slower growth rate, lower Chl *a* content, and reduced O_2_ evolution and consumption (respiration) rates, which are possibly caused by protein over-expression and the resulting metabolic burden in this mutant (**Table 2**). Interestingly, the SZ009 strain showed further reductions in growth rate and O_2_ evolution rate, but had similar Chl *a* contents and O_2_ consumption (respiration) rate when compared to the SZ008 strain (**Table 2**).

### Metabolic Profiling of Glutamate Decarboxylase Expression Strains

**FIGURE 6:**
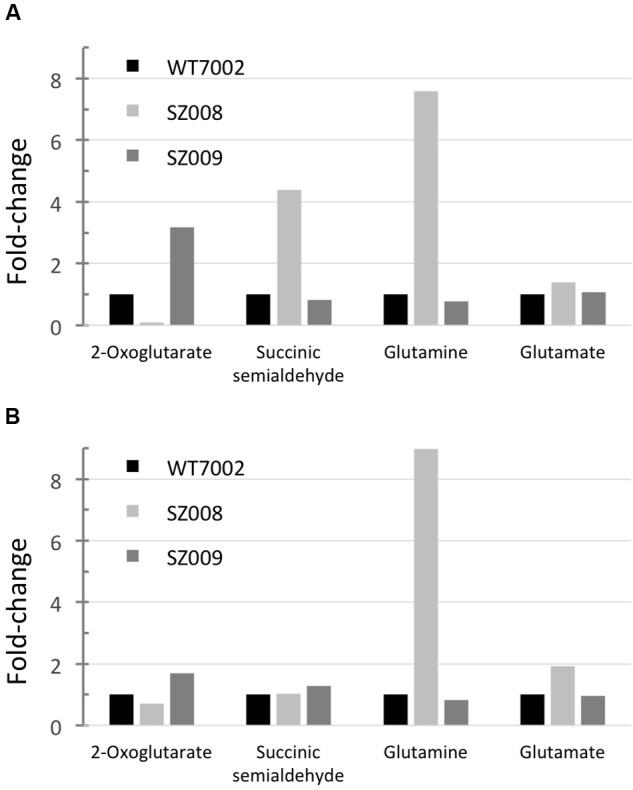
**Metabolites profiling of GABA shunt mutant strains.** Relative metabolites concentrations in strains SZ008 and SZ009, which had been grown under photoautotrophic conditions **(A)** or dark aerobic conditions **(B)**. Relative concentrations for each metabolite in wild-type *Synechococcus* 7002 (WT7002) under these growth conditions were set to 1 unit for comparison.

Metabolic profiling of strains SZ008 and SZ009 described above was performed as described in the Materials and Methods. In the SZ008 strain, the 2-OG level decreased to about 10% of the level in WT7002, while the SSA pool was ∼4.4-fold higher than that in WT7002 under photoautotrophic growth conditions. In addition, glutamine and glutamate concentrations were also higher (7.8- and 1.4-fold, respectively) in strain SZ008 than in the WT7002 strain. These results indicated that 2-OG was indeed directed into the GABA shunt, which produced elevated levels of glutamine, glutamate and SSA (**Figure 6A**).

In the SZ009 strain, 2-OG accumulated to a higher level (3.2-fold) than in the WT7002 strain, while SSA, glutamine and glutamate remained at nearly the same levels as in the WT7002 strain under photoautotrophic conditions. Additionally, it was shown that the SSA content was 50% lower than in the SZ001 mutant strain (**Figure 2B**). Therefore, it is clear that in the SZ009 strain, in which 2-oxoglutarate decarboxylase is absent, the heterologously expressed GadA enables 2-oxoglutarate to be converted to SSA through the reactions of the GABA shunt. However, the reconstructed GABA shunt is less efficient than 2-oxoglutarate decarboxylase in converting 2-OG to SSA, because 2-OG accumulated to a significantly higher level in strain SZ009 (**Figure 6A**).

After these two strains were incubated under dark aerobic conditions for 3 h, the SSA concentration in strain SZ008 was similar to that in strain WT7002. However, a higher (1.7-fold) amount of 2-OG was present in the SZ009 strain, while the 2-OG concentration in the SZ008 strain decreased to ∼70% of the level in WT7002 (**Figure 6B**). These metabolite changes imply that the heterologous GABA shunt is not as efficient as the native 2-OGDC/SSADH bypass in converting 2-OG to SSA in *Synechococcus* 7002, although this could of course result from the heterologous origin and production of GadA_6803_.

## Discussion

These data from this study reveal that during photoautotrophic growth, the TCA cycle functions primarily as a branched pathway for the production of the two essential precursor metabolites, 2-OG and oxaloacetate. Comparison of pool sizes of SSA in WT7002 and the two single-gene knockout mutants (SZ001 and SZ002), which interrupt the 2-OGDC/SSADH bypass route, demonstrate this. When cells respire in the dark, SSA accumulated when SSADH (SsaD) was missing, and SSA levels decreased when OgdA (2-OGDC), the enzyme that produces SSA, was missing. SSA did not accumulate in the SZ002 mutant strain under photoautotrophic conditions, indicating that the SSA route is less active under photoautotrophic conditions than under dark aerobic respiratory conditions. The operation of the TCA cycle as a branched pathway under photoautotrophic conditions is reasonable because cyanobacteria that are fixing CO_2_ should not be simultaneously be oxidizing acetyl units to CO_2_ for ATP production, which can be obtained more efficiently from photosynthetic electron transport. The metabolic importance of the SSA route during respiratory metabolism is demonstrated by the metabolite data in **Figure 4** and further shown by the 2- and 4-fold increases in the relative abundance of transcripts for *ogdA* (SynPCC7002_A2770) and *ssaD* (SynPCC7002_A2771), respectively, under dark aerobic conditions (Ludwig and Bryant, 2011).

Under photomixotrophic conditions, ^13^C-glycerol was rapidly incorporated into SSA. The M_4_/M_0_ ratio (**Figure 2A**) indicates that at least 20% of the carbon molecules in SSA were labeled within 40 min. This observation demonstrates that the SSA route is a major route for converting 2-OG into downstream metabolites. This result contrasts with a ^13^C-metabolic flux study in *Synechocystis* 6803, in which flux through the SSA route was found to be minimal under photomixotrophic conditions (You et al., 2014). One possible explanation for the decreasing percentage of ^13^C-labeled SSA under dark conditions is that there was an increased influx of ^12^C-labeled carbon originating from glycogen consumption in the dark. ^12^C-labeled carbon from glycogen entering the TCA cycle might have diluted the percentage of ^13^C in the total SSA pool.

Deletion of the OgdA/SsaD bypass route may reduce the availability of some unknown metabolite(s) that are not essential but could be beneficial to the growth of *Synechococcus* 7002. The knockout mutants of the OgdA/SsaD bypass route (SZ001 and SZ002) exhibit reduced photoautotrophic growth rates as observed previously (Zhang and Bryant, 2011) and as confirmed again in this study (**Table 2**). Both the reduced Chl *a* contents and oxygen evolution rates of these knockout mutants appear to be responsible for the reduced photoautotrophic growth. These results demonstrate that prior flux balance analysis (FBA) simulations are incorrect in predicting flux to be negligible through the OgdA/SsaD bypass in cyanobacteria under photoautotrophic conditions (Knoop et al., 2013; Vu et al., 2013; Hendry et al., 2016).

The succinate pool size of the SZ007 mutant was similar to that of the WT7002, which contradicts our expectation that this quadruple knockout mutant should not produce any succinate, because both the SDH and the OgdA/SsaD bypass routes are blocked in this mutant. Three TCA cycle variants produce succinate as an intermediate metabolite: (1) the OgdA/SsaD bypass route; (2) the GABA shunt; and (3) the glyoxylate cycle (Steinhauser et al., 2012). However, proteins required to complete the GABA shunt, namely GadA, and the glyoxylate shunt, isocitrate lyase and malate synthase, are all absent in *Synechococcus* 7002 (Zhang and Bryant, 2015). Thus, there must be yet another route to produce succinate in *Synechococcus* 7002. Although, it is well-known that 2-OGDH is absent in all cyanobacteria, succinyl-CoA synthase (encoded by SYNPCC7002_A0890 and SYNPCC7002_A0891) could still convert succinyl-CoA into succinate. Succinyl-CoA can potentially be produced from the degradation pathways for valine, leucine and isoleucine^1^, although not all of the genes involved in these pathways are annotated yet in *Synechococcus* 7002. Pool sizes of succinate and malate were significantly reduced in the SZ006 deletion mutant (**Figure 4**), which supports this hypothesis. Nonetheless, even when the reductive (SDH) and oxidative (OgdA + SsaD) branches of the TCA cycle were blocked, the intracellular succinate concentration in the quadruple mutant strain remained at a comparable level to that in the WT7002 (**Figure 4**). After searching through the metabolic network of *Synechococcus* 7002, we found that L-aspartate oxidase (encoded by SYNPCC7002_A0301) catalyzes the conversion of L-aspartate to iminoaspartate, using O_2_ or fumarate as the electron acceptor. This biochemical reaction yields H_2_O_2_ or succinate, depending on the electron acceptor used (Bossi et al., 2002). A more quantitative fluxomic analysis will be needed to identify the flux contributions of SucCD and L-aspartate oxidase routes to the succinate pool in *Synechococcus* 7002 under both photoautotrophic and dark aerobic respiratory conditions.

The enzymatic and metabolic characterization of the GABA shunt in this study helps to clarify the occurrence of this pathway in cyanobacteria. From the point of view of FBA, the GABA shunt is stoichiometrically identical to the recently discovered TCA cycle variant using OgdA and SsaD. In principle, both variants should result in identical biomass yields if the regulation were identical. However, FBA indicates that the biomass yield using these two pathways is lower than for the conventional cycle using 2-OGDH under respiratory metabolism (Shastri and Morgan, 2005; Knoop et al., 2013). FBA also predicts that autotrophic growth is similarly reduced when metabolites are forced through the 2-OGDH complex or the 2-OGDC/SSADH bypass but not through the GABA shunt. Based on these findings, it has been suggested that the GABA shunt may be an evolutionarily favorable solution to completing the TCA cycle (Nogales et al., 2012). This may also be the reason for the existence of the GABA shunt in some *Prochlorococcus* and marine *Synechococcus* species that lack the 2-OGDC/SSADH bypass. However, our data demonstrate that the GABA shunt is actually less efficient in converting 2-oxoglutarate to SSA in the SZ008 strain of *Synechococcus* 7002. One possible reason might be that heterologous enzyme activity of the expressed glutamate decarboxylase from *Synechocystis* 6803 is not optimal in *Synechococcus* 7002.

Compared to the wild-type, 2-OG levels decreased dramatically in the strain expressing *gadA*, and inactivation of *ogdA* caused 2-OG levels to increase about threefold under photoautotrophic growth conditions. The 2-OG levels might have decreased in SZ008 because there is a second pathway for the consumption of this intermediate in strain SZ008. Deletion of *ogdA* in SZ009 eliminates a major route for 2-OG conversion to SSA, which led to an increase in the 2-OG level in those cells. This explanation is supported by an increase in SSA levels when there are two routes for its synthesis, but SSA levels in SZ009 decreased slightly when OgdA was missing under photoautotrophic conditions. In the dark, 2-OG levels showed much smaller differences and no accumulation of SSA was observed in strain SZ008. Glutamate levels were similar in the WT7002 and SZ009 strains but increased slightly in SZ008. The large increase in glutamine in SZ008 in both the light and the dark is more difficult to explain, but it seems unlikely to be directly related to the introduced GadA because the gene encoding this enzyme is present in both SZ008 and SZ009. The glutamine levels likewise are not correlated with the levels of SSA or glutamate. Instead, this increase seems more likely to be due to currently unexplained kinetic or regulatory effects that might be associated with the lower 2-OG levels in the strain overexpressing the *gadA* gene (SZ008). One possibility is that glutamine synthetase remains active, but the low levels of 2-OG do not allow 2-OG:glutamine amidotransferase (GOGAT) to convert glutamine efficiently into glutamate. Because cells are not nitrogen limited and have a lower 2-OG level, glutamine might accumulate. An explanation for the accumulation of glutamine will require further studies that should probably include transcriptional profiling of these strains.

Genes encoding enzymes for the GABA shunt can be found in the genomes of some cyanobacteria (e.g., *Synechocystis* sp. PCC 6803, *Nostoc* sp. PCC 7107, *Prochlorococcus marinus* str. MIT 9303 and *Synechococcus* sp. RCC307). However, *Prochlorococcus marinus* str. MIT 9303 and *Synechococcus* sp. RCC307 lack the OgdA/SsaD (2-OGDC/SSADH) bypass, and many other *Prochlorococcus* and marine *Synechococcus* species seemingly lack the GABA shunt as well as the 2-OGDC/SSADH bypass. These organisms either have yet another alternative bypass or must have a branched (i.e., interrupted) TCA cycle. Similarly, many other cyanobacteria (e.g., *Synechococcus* 7002) apparently lack the glutamate decarboxylase needed to complete the GABA shunt. Thus, unlike the SSA bypass, the GABA shunt seems to be present in only a few cyanobacteria (Xiong et al., 2014). Considering the mosaic distribution of the complete TCA cycle, the GABA shunt, as well as the glyoxylate cycle in cyanobacteria, the presence of alternative TCA pathways suggests that different options exist for reversible regulation by metabolites. For example, in the case of the GABA pathway, regulation of TCA cycle and hence redox energy production should be possible by nitrogen availability via the shared glutamate/glutamine intermediates.

## Conclusion

Cyanobacteria collectively have four different TCA cycle variants: (1) an open, branched-chain pathway; (2) the OGDC/SSADH bypass; (3) the glyoxylate cycle; and (4) the GABA shunt (Zhang and Bryant, 2014). The sporadic distribution of most of these variants among the highly diverse cyanobacteria, as well as the observation that most cyanobacteria have no more than two of these variants, suggests that these variations reflect specialized responses to the specific environmental niches and must confer important metabolic capabilities upon the organisms that possess them. However, as demonstrated here, it is possible to introduce these variants into strains to modify the metabolic capabilities in ways that could be exploited for biotechnological purposes.

## Author Contributions

SZ constructed and characterized mutants and wrote the manuscript; XQ developed the analytical procedure for detecting succinic acid semialdehyde, analyzed the metabolites in the mutant strains, and assisted in writing the manuscript. SC assisted in the analysis of the mutants. GD and DB supervised the research and participated in writing the manuscript.

## Conflict of Interest Statement

The authors declare that the research was conducted in the absence of any commercial or financial relationships that could be construed as a potential conflict of interest.

## Footnotes

1 http://www.genome.jp/kegg-bin/show_pathway?syp00020
