# Supplementary material for: Natural and Synthetic Variants of the Tricarboxylic Acid Cycle in Cyanobacteria: Introduction of the GABA Shunt into Synechococcus sp. PCC 7002
Source: Front Microbiol. 2016 Dec 9;7:1972. doi: 10.3389/fmicb.2016.01972 (PMC5160925; doi:10.3389/fmicb.2016.01972)
Supplement: Supplementary file 1 [file Presentation_1.PDF]

Supplement Materials for Zhang et al., *Frontiers in Microbiology*
Figures S1 and S2 and Table S1

Natural and synthetic variants of the tricarboxylic acid cycle in cyanobacteria: introduction of the GABA shunt into *Synechococcus* sp. PCC 7002

Shuyi Zhang,[1,a] Xiao Qian,[2,a] Shannon Chang,[2] G. Charles Dismukes,[2,3] and Donald A. Bryant[1,4]*

[1]Department of Biochemistry and Molecular Biology, The Pennsylvania State University, University Park, PA 16802, USA; [2]Waksman Institute of Microbiology and [3]Department of Chemistry & Chemical Biology, Rutgers, the State University of New Jersey, Piscataway, NJ 08854; and [4]Department of Chemistry and Biochemistry, Montana State University, Bozeman, MT 59717 USA

[a] These authors contributed equally to this study.

***Correspondence:** Dr. Donald A. Bryant, 403C Althouse Laboratory, Department of Biochemistry and Molecular Biology, The Pennsylvania State University, University Park, PA 16802 USA. Phone; 814-865-1992; Fax: 814-863-7024; E-mail: dab14@psu.edu

Supplemental Figure S1

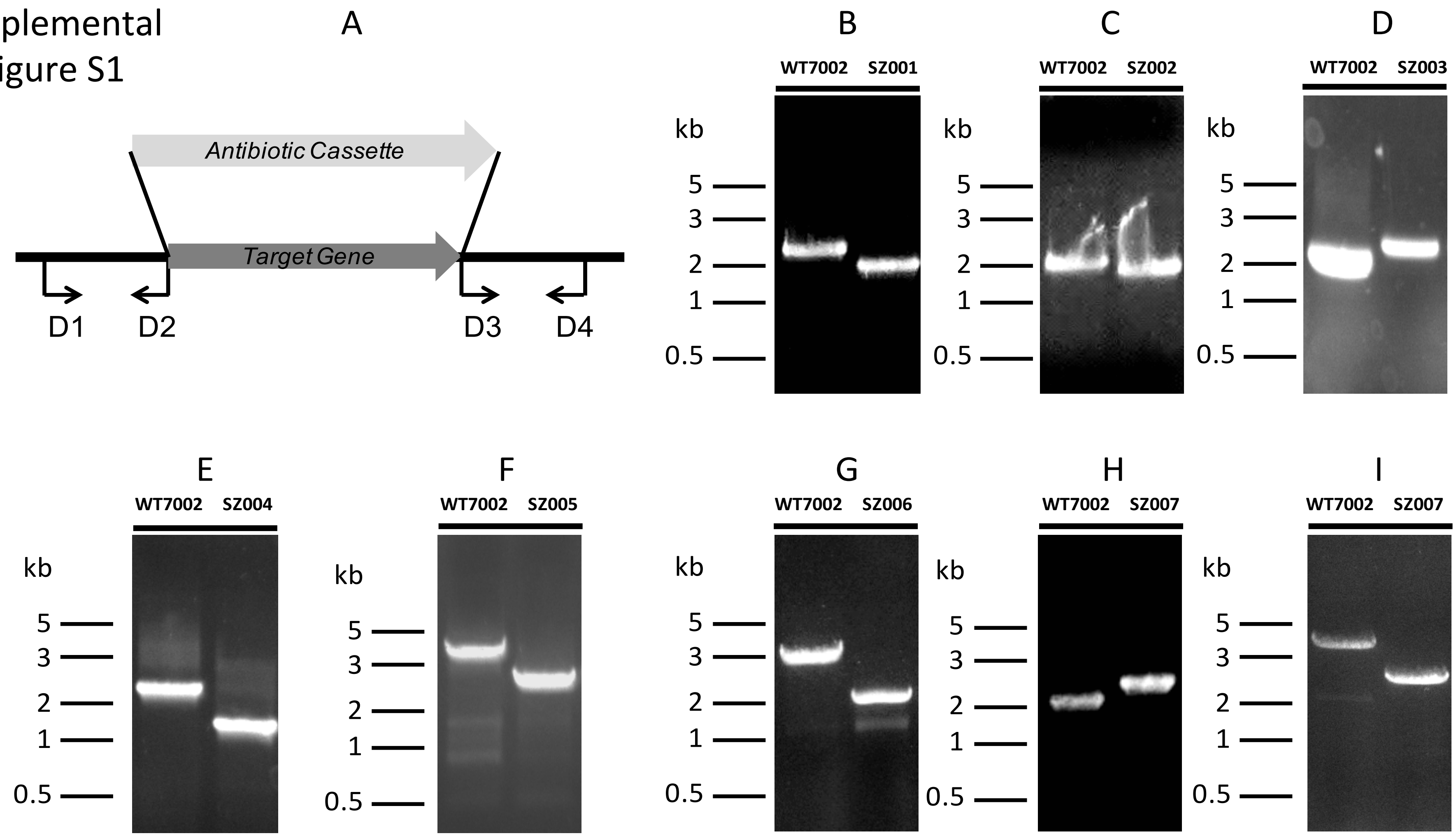

Supplemental FIGURE S1. Scheme for mutant construction and verification of the TCA cycle mutants. A. Upstream and downstream flanking regions for a gene of interest were amplified using primer pairs D1-D2 and D3-D4, and these fragments were ligated to the antibiotic cassette. The resulting construction was transformed into *Synechococcus* sp. PCC 7002 to generate fully segregated mutants based on homologous recombination. (**B to I)** Agarose gel electrophoretic analysis of amplicons from PCR reactions using primers D1 and D4 and template DNA derived from wild type (WT7002) or mutant strains SZ001 (**B**), SZ002 (**C**), SZ003 (**D**), SZ004 (**E**) and SZ006 (**G**), verifying the complete segregation of alleles of the corresponding genes. Additionally, deletion of the operon of *ogdA* (SynPCC7002_A2770) and *ssaD* (SynPCC7002_A2771) in strain SZ005 (**F**), and deletions of *sdhB* (SynPCC7002_A1094) (**H**) as well as the operon of *sucCD* (SynPCC7002_A0890 and SynPCC7002_A0891) (**I**) in the SZ007 mutant strain were also verified using the same method.

Supplemental Figure S2

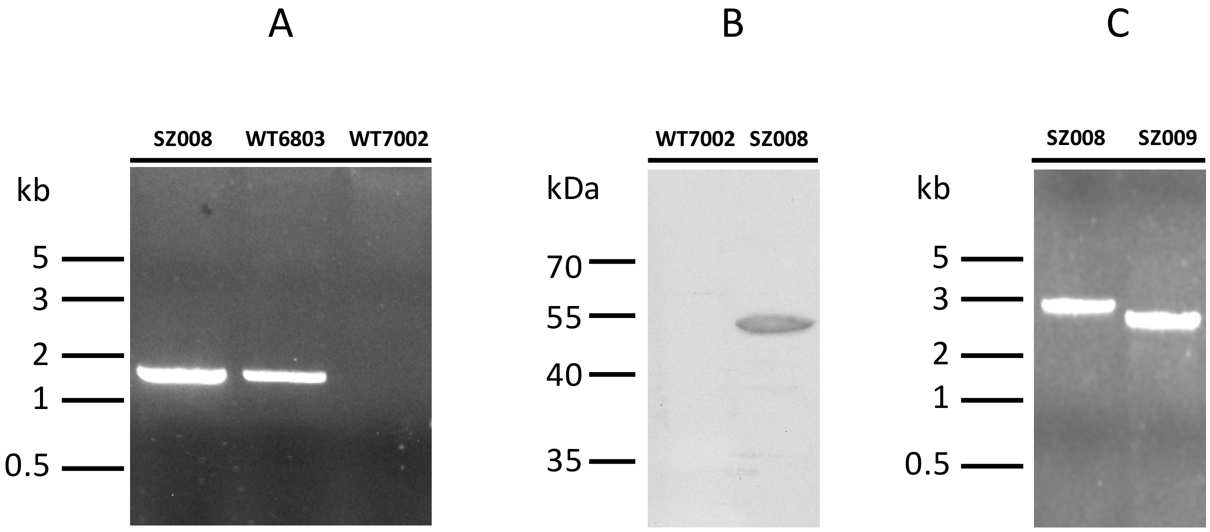

Supplemental Figure S2. Verification of the constructed GABA shunt mutant strains. (A) Verification of the presence of glutamate decarboxylase gene. The template DNA was derived from wild-type *Synechococcus* 7002 (WT7002), from wild-type *Synechocystis* 6803 (WT6803), and from the recombinant strain SZ008, which has the *sll1641* gene from *Synechocystis* 6803 inserted in plasmid pAQ1-Ex. (**B**) Immunoblotting of whole-cell extracts of WT7002 as well as SZ008 strain with antibodies to the His_{10}-tag, demonstrating the successful expression of glutamate decarboxylase. (**C**) Agarose gel electrophoretic analysis of amplicons from PCR reactions using primer set A2770D1-A2770D4, verifying the fully deletion of SYNPCC7002_A2770, using DNA templates from strains SZ008 or SZ009.

Table S1. Primers used in this study

Name	Sequence (5' to 3')
A2770D1	TGCAGTGGATCCGGACGATCGCCT
A2770D2	ATGTAAAAGCTTGTCGTACTGGGG
A2770D3	GCCTTGGTCAGTGAGCCAATTTTG
A2770D4	AATGGTCAATCTTGTATTGCGGCG
A2771D1	AATCGAAGTCGTCTTTTTACCCGCCGAGCA
A2771D2	AATGATGGATCCAACTCTCTCCTTTGCCGC
A2771D3	TGAATGAAGCTTCCCCCCGACTTTATCCCA
A2771D4	TAGATTTTTTCACGACCATCCCGCTGGAGG
A2569D1	ATAACAGCGATCGCCAATTACGCCCGTGT
A2569D2	CGGCGCTCTAGAAAAACTCAGGGGTTATTC
A2569D3	GCTTAAGCATGCCAGCCCTTCTCAAAGATG
A2569D4	GTCGATTTTATCGCTACCGAAAACCCCGAC
A1094D1	TTGTGAACACTCACCCGATAGGTCGGCTCT
A1094D2	GTCCGTGGATCCCCTCTTTTTGCCACAAAT
A1094D3	GCGCGCAAGCTTAAATGAGGGGGCTTAAAT
A1094D4	CGAAGGCGATCGCCCACTGTCTTTTTTTC
A9091D1	ACCCAAATCTGTTTGAAGCTCAAATCCGCC
A9091D2	GAGCAAAAGCTTTAGCGCAGAGGCAACCTC
A9091D3	ACGACCGGATCCATTTGCACCGCTTAGATC
A9091D4	CTTCGAGGGGGATGTGATATTCCTTGAGCA
GADF	GGCCGGCATATGGTGCATAAAAAAATTGAC
GADR	AATACTGGATCCCTAATGGCTAAAGTGGGA
6803ArgDF	GGTGATCATATGACCTATTCCCCTGTTGTT
6803ArgDR	TTAGATGGATCCTCAAACCAAAGTGGCGAT
7002ArgDF	AGTATTCATATGAGTCCCCAAACGCTACTG
7002ArgDR	AATGGTCAATCTTGTATTGCGGCG
